# Two young athletes with Tetralogy of Fallot—stress echocardiography in therapeutic decision-making: a case report

**DOI:** 10.1093/ehjcr/ytag045

**Published:** 2026-01-24

**Authors:** Melina Winkler, Nuno Duarte, A Graham Stuart, Guido E Pieles

**Affiliations:** Department of Paediatrics and Adolescent Medicine, Division of Pediatric Cardiology, Medical University Graz, Auenbruggerplatz 34/II, 8036 Graz, Austria; Bristol Congenital Heart Centre, University Hospitals Bristol and Weston NHS Foundation, Upper Maudlin Street, Bristol BS2 8BJ, UK; Bristol Congenital Heart Centre, University Hospitals Bristol and Weston NHS Foundation, Upper Maudlin Street, Bristol BS2 8BJ, UK; Department of Athlete Screening and Sports Cardiology, Aspetar Orthopaedic and Sports Medicine Hospital, Sport City Street, Doha, Qatar; Institute of Sport, Exercise and Health, University College London, 170 Tottenham Court Road, London W1T 7HA, UK

**Keywords:** Congenital heart disease, Adolescent athlete, Stress echocardiography, Cardiopulmonary exercise testing, Sports cardiology, Interventricular dyssynchrony, Case report

## Abstract

**Background:**

The surveillance of athletes with congenital heart disease remains challenging despite recent recommendations. Stress echocardiography as a diagnostic tool is not yet part of the routine follow-up but has emerged as an innovative approach to assess cardiac reserve and exercise capacity. It can unmask cardiac exercise pathophysiology and hence inform decision making for repeat interventions in athletes with complex congenital heart disease.

**Case summary:**

This case report describes two athletes with Tetralogy of Fallot who, despite similar conditions, exhibit different cardiovascular risks. Their above average exercise capacity masks early cardiac deterioration, underscoring the limitations of cardiopulmonary exercise testing in assessing myocardial function. Stress echocardiography revealed severe biventricular dyssynchrony in one athlete, playing a major role in decision-making to perform a pulmonary valve replacement (PVR). Post transcatheter PVR, improved left ventricular–right ventricular synchrony was seen at rest and during exercise stress echocardiography.

**Conclusion:**

In athletes with congenital heart disease (CHD), disease worsening might be masked by above-normal exercise capacity, and detailed assessment, including cardiopulmonary exercise testing and exercise echocardiography, might be needed to detect underlying pathophysiology and hence guide the therapeutic approach. Two-strain during exercise echocardiography can be used to quantify cardiac function but also decipher interventricular dyssynchrony in CHD.

Learning points

- In athletes with congenital heart disease, disease progression may be masked by above-normal exercise capacity.
- A comprehensive evaluation, including cardiopulmonary exercise testing and exercise echocardiography, may be required to uncover underlying pathophysiology and guide management.
- Strain analysis during exercise echocardiography can further quantify ventricular function and identify interventricular dyssynchrony in congenital heart disease

**Topic: Issue Section 55: Case Report > Sports and heart disease**

## Introduction

Advances in the diagnosis and long-term survival of congenital heart disease (CHD) have enabled an increasing number of patients to maintain a more active lifestyle and participate in sports. Unlike the sedentary majority, there is an emerging patient subpopulation of young CHD patients training at an elite level. These athletes, however, require thorough diagnostic evaluation to prevent disease complications or cardiac adverse events associated with sports.^1^

In this case report, two athletes with Tetralogy of Fallot (TOF) are presented detailing innovative diagnostic workup following the recent recommendations for athletes over the age of 16 years with CHD.^1–3^ Compared to previous guidelines, which primarily focus on anatomical lesions, the new recommendations emphasize a more functional approach to assessing an athlete’s heart with CHD including comprehensive assessment during exercise. Although the underlying anatomical lesion is important, a functional approach allows for more personalized decision-making tailored to the patient. The following case report highlights the benefits of exercise stress echocardiography in young athletes with repaired CHD.

## Summary figure

**Figure:**
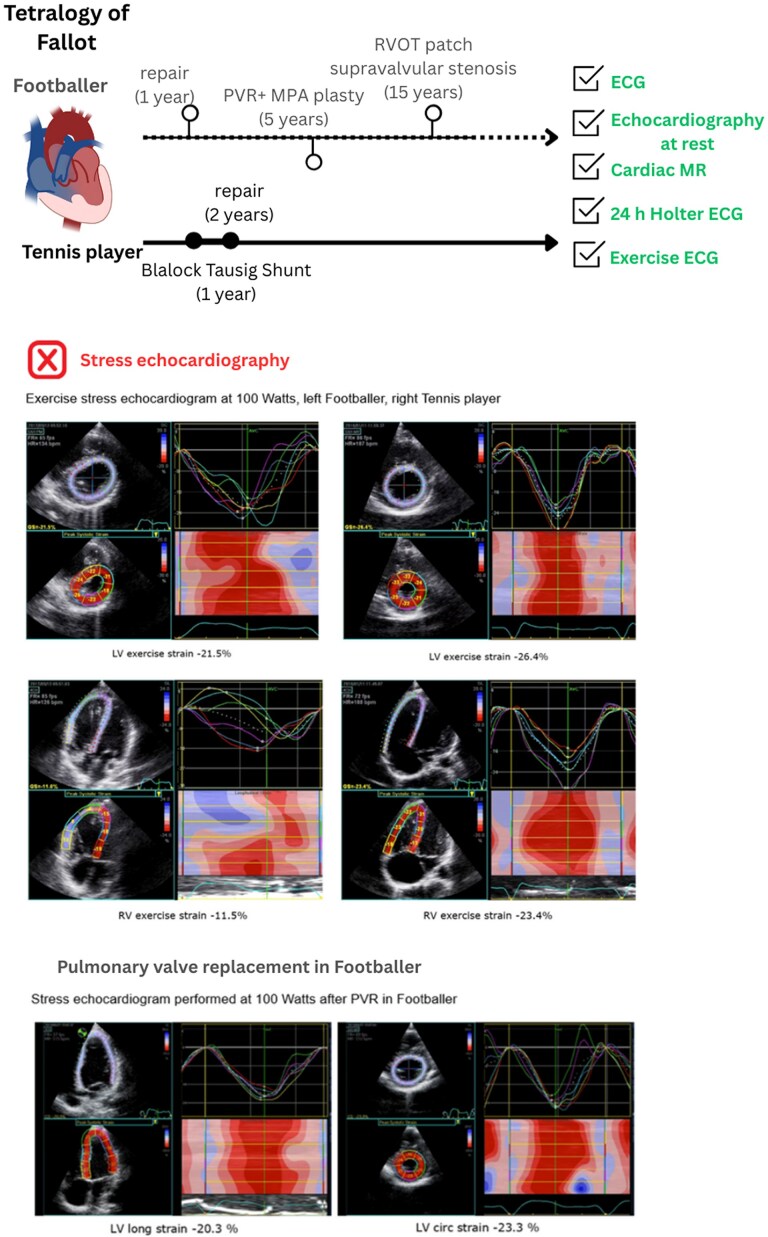


## Case presentation

Two 16-year-old male athletes with TOF and matching characteristics and disease phenotype underwent a routine diagnostic workup. One of the athletes was a UK county-level footballer, while the other qualified for a US sports college scholarship in tennis.

The routine assessment comprised electrocardiogram (ECG) and echocardiography at rest, cardiac magnetic resonance imaging (CMR), cardiopulmonary exercise testing (CPET), and simultaneous stress ECG on an ergometer, as well as a 24-h Holter ECG and additional quantitative exercise stress echocardiography (*Table 1*). On echocardiography, the footballer had an enlarged RV in the apical four-chamber view and the tennis player showed both an enlarged right atrium and RV. LV function was measured within the normal range in both athletes (Supplementary material online, Videos S1–S4). The CMR confirmed the pressure and volume overload of the footballer’s heart, with a pulmonary regurgitation (PR) that was quantified as mild to moderate. Compared to the footballer’s heart, the tennis player had significantly less RV pressure and volume overload. In CPET, the footballer had a peak oxygen consumption (VO_2_peak) above average, although he showed signs of chronotropic insufficiency. The tennis player performed even better. Both results showed good fitness levels especially considering their underlying heart condition.^4^ The stress and Holter ECG showed no arrhythmia, there were only occasionally some monomorphic ventricular extrasystole (VES) originating from the RV outflow tract (RVOT) in the footballer. The QRS complex (QRS) duration was moderately prolonged in the footballer, whereas the tennis player only showed a partial right branch bundle block (*Table 1*).

**Table 1:** Diagnostic workup of the two athletes

Baseline characteristics		
	Footballer	Tennis player
Age (years)	16	16
Weight (kg)	62	71.7
Height (cm)	179	177.5
BMI (kg/m^2^)	19	22
Cardiac diagnosis	TOF, repair (1 year), PVR + MPA plasty [5 years], RVOT patch/supravalvular stenosis (15 years)	TOF, right Blalock Tausig Shunt (1 year), total correction (2 years)
Clinic presentation	Athletic body habitus	Athletic body habitus
	Systolic murmur 2/6	2/6 systolic murmur
	No medication	No medication
Past medical history	No syncope, above average subjective exercise tolerance, but decreasing over last 6 months, no family history of SCD	No syncope, above average subjective exercise tolerance, no family history of SCD
Family history		
**ECG**		
	SR with RBBB	SR with partial RBBB
HR at rest (bpm)	65	62
Resting ECG QRS duration (ms)	160	110
QRS axis (degrees)	+97	+90
	Normal intervals, RVH	Normal intervals, no LVH/RVH
**24-h Holter ECG**		
	SR, monomorphic VES <1%	SR, monomorphic VES <1%
**CPET**		
VO2max (mL/kg/min)	40.2	47
VO2/HR (mL/beat)	16	16
AT/VO2 max (%)	56	61
VE/VO2	28	31
HR max. (bpm)	156	187
RER	1.09	1.1
**Cardiac MR**		
LV-EF (%)	50; 57–77	62; 57–77
LV-EDV (mL/m^2^)	103; 68–112	100; 68–112
LV-ESV (mL/m^2^)	51;16–44	38; 16–44
RV-EF (%)	34; 47–67	64; 47–67
RV-EDV (mL/m^2^)	177; 74–134	116; 74–134
RV-ESV (mL/m^2^)	119; 26–62	42; 26–62
PR (%)	32	17
	No gadolinium delayed enhancement	No gadolinium delayed enhancement
**Echocardiography at rest**		
IVSd (mm)	8	9
LVIDd (mm)	55	47
LVIDs (mm)	35	35
FS (%)	36	30
MAPSE	10.5	17
RVOT vel. (m/s)	4.3	2.9
RVSP (mmHg)	72	35
TAPSE	12.5	11
RVOT peak gradient (mmHg)	37	34
RVSP (mmHg)	75	
**Stress echocardiography—resting strain**		
LV peak systolic longitudinal strain (%)	−15	19.9
LV peak systolic circumferential strain (%)	−18	−24.7
RV peak systolic longitudinal strain (%)	−18	−15
**Stress echocardiography**		
HR max. (bpm)	203	184
LV peak systolic circumferential strain (%; at 100 W/rec. 2 min)	−21.5/−23	−28/−26
LV peak systolic longitudinal strain (%; at 100 W/rec. 2 min)	−19/−20	
RV peak systolic longitudinal strain (%; at 100 W/rec. 2 min)	−11.5/−14	−23.4/−20
**Exercise ECG**		
	No ischaemic or repolarization ST changes, normal shortening of QT interval, max HR 203 bpm, reduced frequency of VES, no VT, or SVT; frequent monomorphic ventricular ectopics	No ischaemic or repolarization ST changes, normal shortening of QT interval, max HR 203 bpm, no ectopics, or arrhythmic activity

All investigations were performed contemporaneously within 3 months in both athletes.

ECG, electrocardiogram; EDV, end diastolic volume; EF, ejection fraction; ESV, end systolic volume; FS, fractional shortening; HR, heart rate; IVDd, intraventricular septal diameter diastolic; IVSd, intraventricular septal diameter systolic; LV, left ventricle/left ventricular; LVH, left ventricular hypertrophy; MAPSE, mitral annular plane systolic excursion; MPA, main pulmonary artery; PR pulmonary regurgitation; PVR, pulmonary valve replacement; RBBB, right bundle branch block; RER, respiratory exchange ratio; RV, right ventricle/right ventricular; RVH, right ventricular hypertrophy; RVOT, right ventricular outflow tract; SCD, sudden cardiac death; SP, systolic pressure; SR, sinus rhythm; TAPSE, tricuspid annular plane systolic excursion; TOF, Tetralogy of Fallot; VES, ventricular extrasystole.

To assess exercise-related symptoms, cardiac function, RV pressure, and myocardial reserve, both athletes underwent stress echocardiography on a semi-supine (45°) cycle ergometer. The protocol included 3-min stages with 50-W increments for steady-state echocardiographic data acquisition as prescribed previously in adolescent athletes.^5–7^ Focused 2D strain echocardiography was performed at rest, during unloaded pedalling, at 50 W, 100 W, and 2 min into recovery. The gas exchange threshold was expressed as a percentage of VO₂peak. Myocardial reserve was defined as the difference in mean peak systolic strain between baseline and each stage. Metabolic parameters were analysed concurrently during simultaneous CPET. Only images with frame rates between 60 and 100 frames per second were included to ensure adequate temporal resolution for strain analysis at higher heart rates. A minimum of three cardiac cycles were recorded per stage to include at least one expiratory cycle, visually confirmed for optimal image quality prior to strain analysis. In this protocol, imaging was performed according to workload rather than target heart rate, in line with previously published methodology by Pieles *et al*.^6,7^ Myocardial strain is known to be heart rate-dependent during exercise, consistent with findings by Cifra *et al*.^8^ in a healthy paediatric cohort and Forsey *et al*.^9^ in children with congenital heart disease.^8,9^ This approach accounts for the observed differences in heart rate during image acquisition.

**Figure 1:**
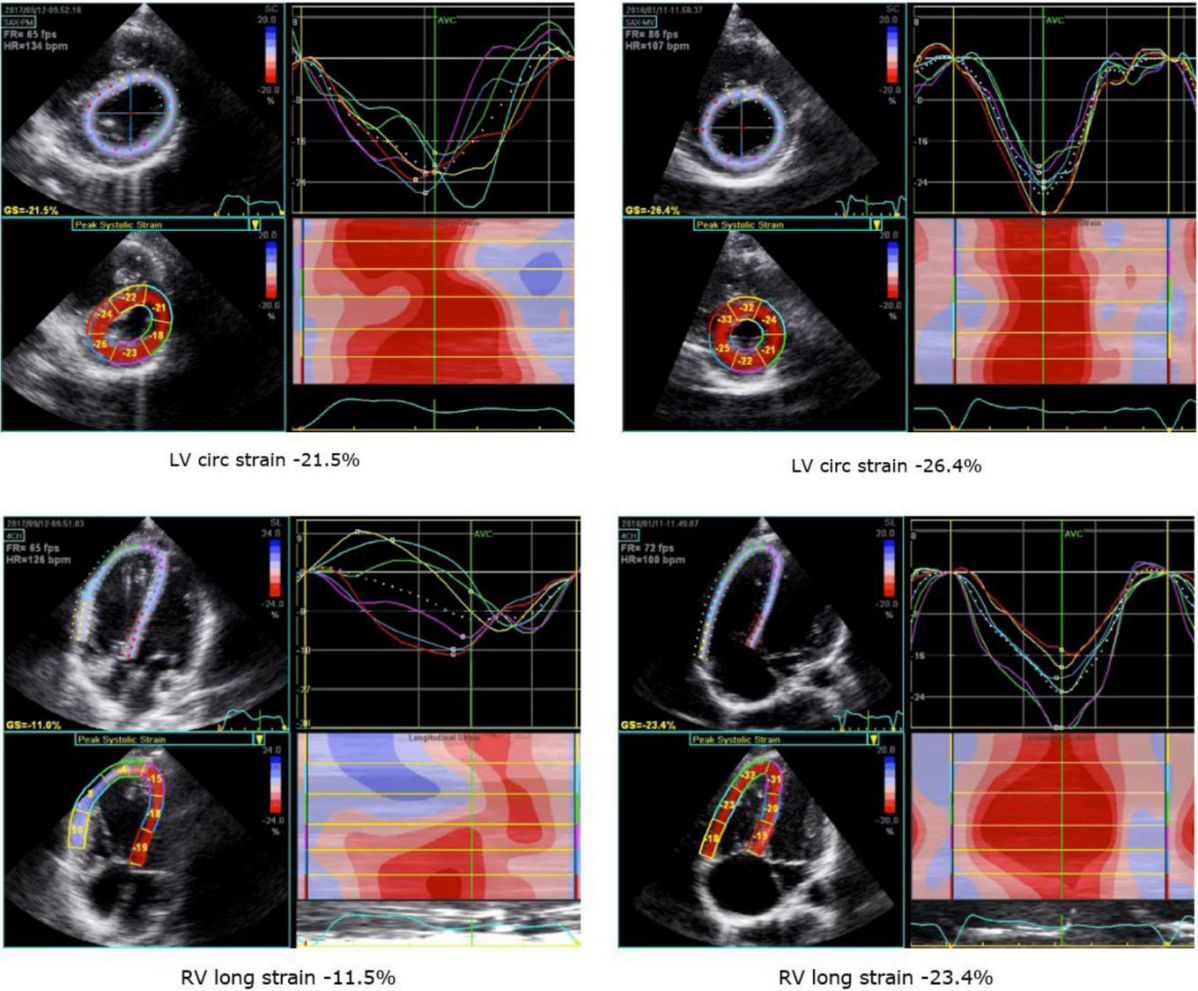
Exercise stress echocardiogram at 100 W, left footballer, and right tennis player.

**Figure 2:**
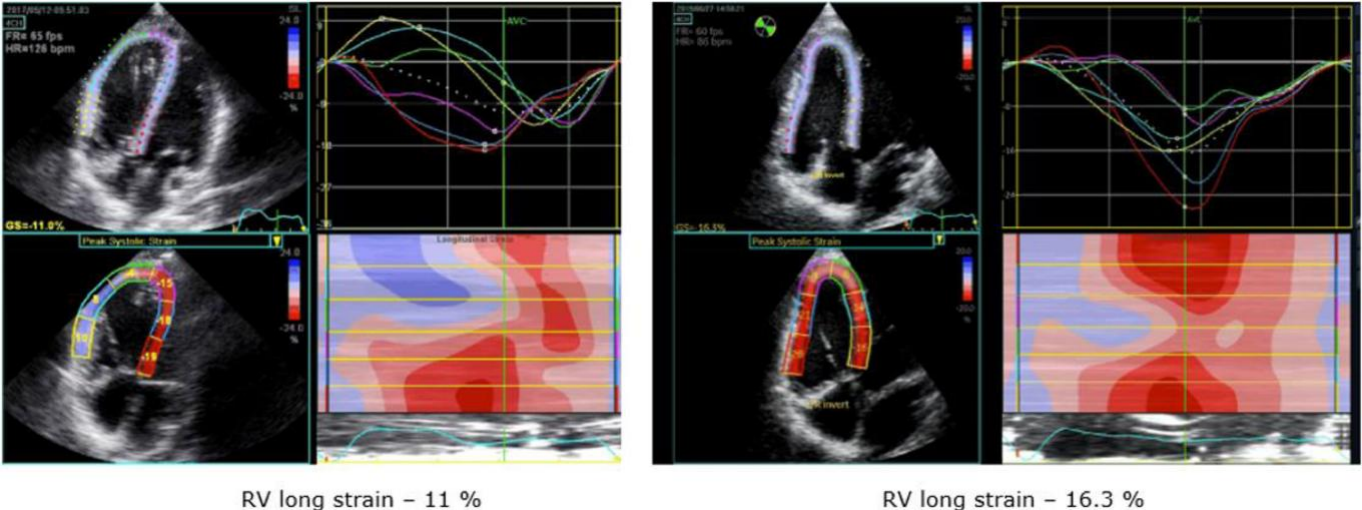
Stress echocardiogram performed at 50 W before and 4 months after pulmonary valve replacement in footballer.

**Figure 3:**
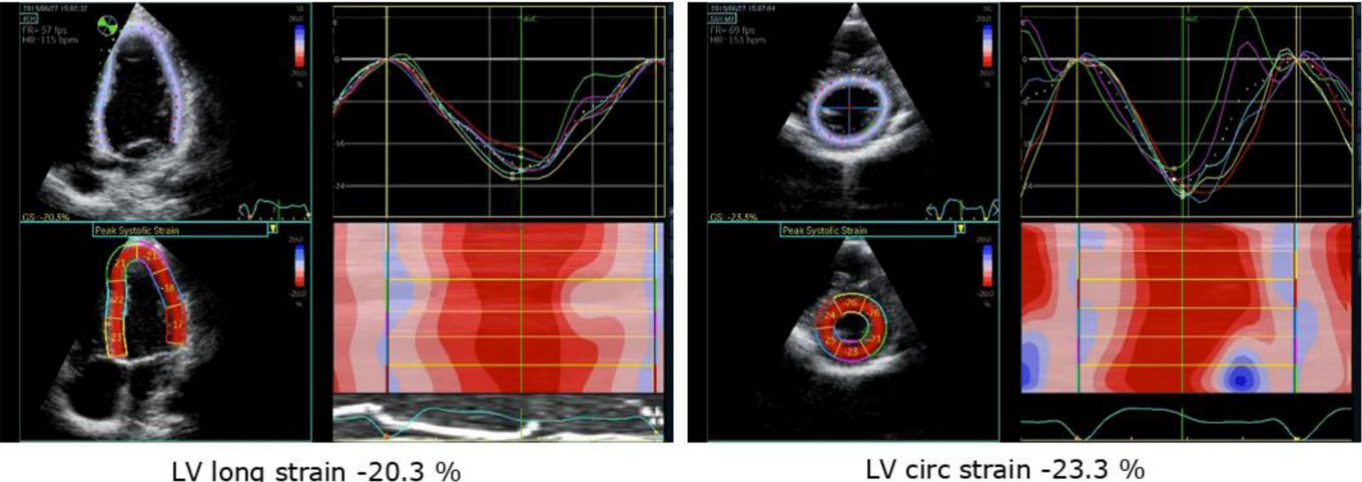
Stress echocardiogram performed at 100 W 4 months after pulmonary valve replacement in footballer.

At a workload of 100 W, the footballer’s RV exhibited significant pressure and volume overload, accompanied by severe LV–RV dyssynchrony as measured by 2D strain (*Table 1*, Supplementary material online, *Videos S5, S6, S9* and *S10*). The mild RV impairment observed at rest worsened during exercise, with RV longitudinal strain decreasing from −18% at rest to −11.5% at 50 W, showing a decompensating RV, that also showed significant dilation. Notably, ventricular–ventricular interaction led also to a consecutive decline in LV function, with LV circumferential strain decreasing to −21.5%, falling below published reference values during exercise,^6,8^ showing a poor LV myocardial reserve. Although the tennis player displayed increased RV diameters at rest, he demonstrated good myocardial reserve with synchronized biventricular function and near-normal strain values at 100 W (*Table 1, Figure 1*, Supplementary material online, *Video S7, S8 and S10*). Considering the severe dyssynchrony during exercise resulting from a pressure-loaded RV as a cause of biventricular dysfunction, resulting in increasing exercise symptoms, the footballer underwent transcatheter pulmonary valve replacement (PVR). Post catheter intervention, both RV longitudinal and LV circumferential strain patterns showed significant improvement, leading to a more synchronized biventricular response to exercise as early as 4 months after the PVR (*Figure 1–3*, Supplementary material online, *Videos S11* and *S12*).

## Discussion

Sports participation in patients with CHD remains an evolving and complex topic. Therapeutic decision-making for athletes with CHD poses significant challenges, given the wide spectrum of haemodynamic consequences and prognostic outcomes associated with different congenital defects, even in individuals with the same diagnosis. This case report aims to highlight the role that exercise echocardiography can play in individual diagnosis and decision-making.

Exercise assessment is at the core of the most recent European Association of Preventive Cardiology and Assoociation for European Paediatric and Congenital Cardiology recommendations on the assessment of athletes with CHD and while CPET is the current gold standard for risk stratification and assessment of exercise capacity, it has inherent limitations.^1^ CPET primarily evaluates oxygen delivery, driven largely by increased cardiac output, but does not directly assess myocardial function in response to exercise stress. Consequently, it provides limited insight into cardiac reserve and relies on indirect inferences.^7^ Furthermore, as our case highlights, an above-average and normal exercise capacity due to athletic training measured during CPET may indeed conceal early signs of cardiac deterioration, as both athletes’ VO_2_peak were above the 80th centile for TOF patients.^4^ Combining CPET with stress echocardiography can overcome this limitation, as shown here, and may reveal myocardial exercise pathology, as the cause of symptoms and help implement aetiology-based therapy.^6–10^

PVR is performed to mitigate the adverse effects of chronic RV volume and pressure overload, yet the decision on when to intervene is complex and the optimal timing of PVR in asymptomatic or mildly symptomatic TOF patients remains uncertain.^11^ Following interventional PVR in the football player, both RV longitudinal strain and global LV strain during exercise showed significant improvement. This can be attributed to the relief of the RVOT obstruction, which improved the previously significant RV–LV dyssynchrony, which not only impaired RV function but also adversely affected LV performance via interventricular dyssynchrony,^12^ as shown by the specific RV and LV strain pattern in this case. In TOF patients, interventricular dyssynchrony—reflected in the RV–LV delay due to the consequences of chronic RV pressure loading—is associated with prolonged QRS duration, reduced RV–EF, and consequently, diminished VO₂peak.^13^ Establishing this precise pathophysiology in our case using exercise echocardiography led to timely intervention by catheter PVR and follow-up exercise echocardiography confirmed improvement in RV and LV function and synchrony (*Figure 3*).

In summary, in athletes with CHD, disease worsening might be masked by above-normal exercise capacity and detailed assessment including CPET and exercise echocardiography might be needed to detect underlying pathophysiology and hence guide the therapeutic approach. Two-strain during exercise echocardiography can be used to quantify cardiac function but also decipher interventricular dyssynchrony in CHD.^14^

## Lead author biography


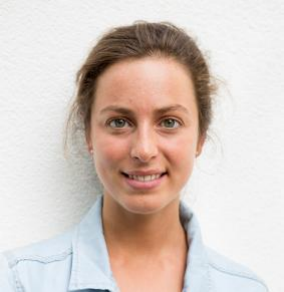


Dr. M. Winkler is a senior resident in paediatric cardiology at the Medical University of Graz, Austria. Her clinical interests include congenital heart disease, with a particular focus on sports cardiology.

## Supplementary Material

ytag045_Supplementary_Data

## Data Availability

All available data relevant to this case report have been included within the manuscript.

## Notes

**Consent:** The authors confirm that written consent for submission and publication of this case report including image(s) and associated text has been obtained from the patients in line with COPE guidance.
